# Relationship of α-fetoprotein levels and development of hepatocellular carcinoma in hepatitis C patients with liver cirrhosis

**DOI:** 10.3892/etm.2012.709

**Published:** 2012-09-17

**Authors:** NAOTA TAURA, SACHIKO FUKUDA, TATSUKI ICHIKAWA, HISAMITSU MIYAAKI, HIDETAKA SHIBATA, TAKUYA HONDA, TOHEI YAMAGUCHI, YOKO KUBOTA, SHINJIRO UCHIDA, YASUHIRO KAMO, EMI YOSHIMURA, HAJIME ISOMOTO, TAKEHIRO MATSUMOTO, FUMINAO TAKESHIMA, TAKUYA TSUTSUMI, SHOTARO TSURUTA, KAZUHIKO NAKAO

**Affiliations:** 1 Department of Gastroenterology and Hepatology, Graduate School of Biomedical Sciences, Nagasaki University, Nagasaki 852-8501;; 2 Department of Gastroenterology and Hepatology, Nagasaki Municipal Hospital, Nagasaki 850-8555; 3 Department of Gastroenterology and Hepatology, Japanese Red Cross Nagasaki Genbaku Hospital, Nagasaki 852-8511, Japan

**Keywords:** hepatocellular carcinoma, hepatitis C virus, α-fetoprotein

## Abstract

α-fetoprotein (AFP) is a tumor marker of hepatocellular carcinoma (HCC) and has also been reported to reflect the effectiveness of long-term low-dose interferon (IFN) therapy in hepatitis C virus (HCV)-infected patients with chronic liver disease. The correlation between AFP levels and the incidence of HCC has been discussed over a long period. We investigated whether high levels of AFP at the time of diagnosis were associated with an increased incidence of HCC in patients with HCV. A total of 107 HCV patients with liver cirrhosis without other risks were evaluated for the predictive value of non-invasive risk factors for HCC, including age, gender, alcohol intake, aspartate and alanine aminotransferase levels, bilirubin, albumin, platelet count and AFP levels at study entry, as well as the IFN therapy received. During the follow-up period, HCC developed in 68 (63.6%) patients. Kaplan-Meier estimates were made to assess the cumulative risk of HCC. The 10-year cumulative incidence rate of HCC was 80%. Cox regression analysis was performed on several variables, including age, gender, alcohol consumption, experience of IFN therapy and biochemical parameters. The following factors were identified as exhibiting an increased risk of HCC by univariate analysis: aspartate transaminase (AST) ≥71 IU/l, alanine transaminase (ALT) ≥60 IU/l, AFP ≥6 ng/ml and IFN therapy. Multivariate analysis identified that the AFP level [6–19 ng/ml: hazard ratio (HR), 2.22; P=0.006 and ≥20 ng/ml: HR, 2.09; P=0.003] was an independent and significant risk factor for the development of HCC. A slightly elevated (6–19 ng/ml) AFP level may be a risk factor for HCC in certain cases. By contrast, AFP levels <6 ng/ml indicate a low risk of HCC development in HCV patients with liver cirrhosis.

## Introduction

Primary liver cancer is the most common primary cancer of the liver, accounting for approximately 6% of all human cancers. It is estimated that half a million cases are diagnosed worldwide annually, making primary liver cancer the fifth and ninth most common malignancy in males and females, respectively (1–6). Hepatocellular carcinoma (HCC) accounts for 85–90% of primary liver cancers (7) and the age-adjusted HCC mortality rate has increased in recent decades in Japan (8). Similarly, a trend of increasing rates of HCC has been reported in several developed countries in North America, Europe and Asia (9,10). HCC often develops in patients with liver cirrhosis caused by hepatitis B virus (HBV) or hepatitis C virus (HCV) infection, excessive alcohol consumption or non-alcoholic fatty liver disease. Of the hepatitis viruses that cause HCC, HCV is predominant in Japan (11–14).

α-fetoprotein (AFP) is a tumor marker of HCC and is also reported to reflect the effectiveness of long-term low-dose interferon (IFN) therapy in HCV patients with chronic liver disease (15). The correlation between AFP levels and the incidence of HCC has been discussed over a long period. We investigated whether high levels of AFP at the time of diagnosis were associated with an increased incidence of HCC in patients with HCV.

## Patients and methods

### Study population

Between 1976 and 2010, 107 patients were diagnosed with liver cirrhosis due to HCV infection at the Department of Gastroenterology and Hepatology, Nagasaki University Hospital (Nagasaki, Japan). The diagnosis of liver cirrhosis was based on biopsy and/or clinical findings. Sera were stored at −80°C until they were used for assays. The diagnosis of chronic HCV infection was based on the presence of anti-HCV antibodies (HCV Abs; microparticle enzyme immunoassay; Abbott Laboratories, Chicago, IL, USA) and HCV RNA, as detected by the polymerase chain reaction. The diagnosis of chronic HBV infection was excluded on the basis of the presence of hepatitis B surface antigen (HBsAg; enzyme-linked immunosorbent assay; Abbott Laboratories). Serum AFP was measured using a radioimmunoassay (Abbott Laboratories). The patient alcohol intake histories were obtained from their medical records. Habitual drinking was defined as an average daily consumption of an amount equivalent to 80 g of pure ethanol for a period of >10 years.

### Follow-up of patients and diagnosis of HCC

Following the initial diagnosis of patients with liver cirrhosis and HCV infection, the patients underwent measurement of AFP levels and liver function biochemistry every 1 to 3 months during the follow-up period and ultrasonography (USG) was performed every 3 to 6 months. The diagnosis of HCC was based on imaging techniques, including USG, computerized tomography (CT), magnetic resonance imaging (MRI), hepatic angiography (HAG) and/or liver biopsy. The diagnostic criteria for HCC included confirmative liver biopsy, neovascularization in HAG and/or CT.

The end date of the present study was December 2010, detection of HCC or the time of patient mortality. If a patient had not been monitored in the hospital for >1 year, the patient was considered lost to the follow-up. The median observation period was 3.8 years (IQR, 5.0).

### IFN therapy

During the observation period, 43 (40%) of the 107 patients received IFN monotherapy, PEGylated (PEG)-IFN monotherapy or combination therapy with IFN and ribavirin or PEG-IFN and ribavirin. A sustained virological response (SVR) was defined as the absence of detectable HCV RNA at the end of treatment persisting for >6 months, while a failure to meet these criteria was defined as non-SVR. There were no relapses of viremia in the SVR patients after 6 months.

### Statistical analysis

The HCC development rate was analyzed using the Kaplan-Meier technique and differences in the curves were studied using the log-rank test. Independent risk factors associated with the rate of HCC development were identified using the stepwise method of non-time-dependent Cox regression analysis. Parametric comparisons were performed using analysis of variance (ANOVA). The significance of individual differences was evaluated using the Scheffe test. Data analysis was performed with SPSS version 16.0 for Windows. P<0.05 was considered to indicate a statistically significant result.

## Results

### Clinical features of the studied patients

Patient characteristics at the time of the cirrhosis diagnosis are shown in Table I. There were 54 male (51%) and 53 female (49%) cirrhosis patients (median age, 62.5 years). Habitual drinkers and diabetic patients were 10% (11 of 107) and 44% (41 of 107) of all cases, respectively. Child-Pugh grade A was recorded in 52% (56 of 107) of patients, grade B in 41% (44 of 107) and grade C in 7% (7 of 107). Of the studied patients, 40% (43 of 107) underwent IFN therapy and 60% (64 of 107) were followed closely without receiving IFN treatment. The proportion of IFN-treated patients exhibiting an SVR was 25.6% (11/43). The patients were classified into 3 categories according to the level of AFP. The AFP levels were <6 ng/ml in 34 (32%) patients, between 6 and 19 ng/ml in 38 (35%) and ≥20 ng/ml in 35 (33%).

### Risk factors for HCC

Cox regression analysis was performed on variables, including age, gender, alcohol consumption, experience of IFN therapy and biochemical parameters. The following factors were identified as exhibiting an increased risk of HCC by univariate analysis: aspartate transaminase (AST) ≥71 IU/l, alanine transaminase (ALT) ≥60 IU/l, AFP ≥6 ng/ml and IFN therapy (Table II). Multivariate analysis identified the etiology of the AFP level [6–19 ng/ml: hazard ratio (HR), 2.22; P=0.006 and ≥20 ng/ml: HR, 2.09; P=0.003] as independent and significant risk factor for the development of HCC (Table III).

### Development of HCC

**Figure 1:**
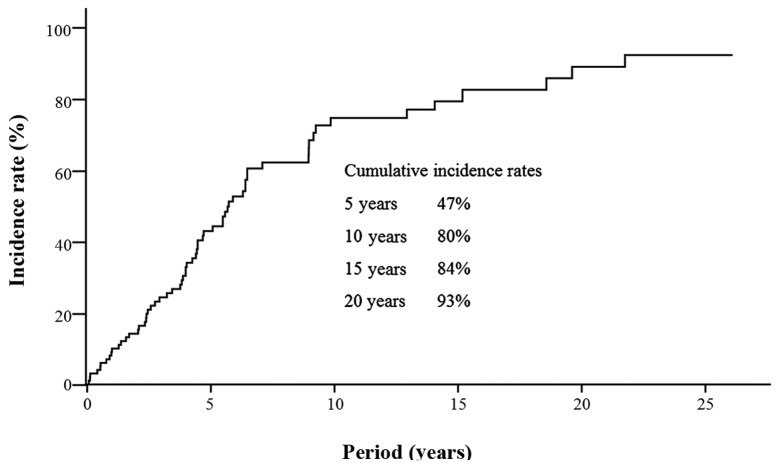
Cumulative incidence rates of hepatocellular carcinoma (HCC) in all patients.

**Figure 2:**
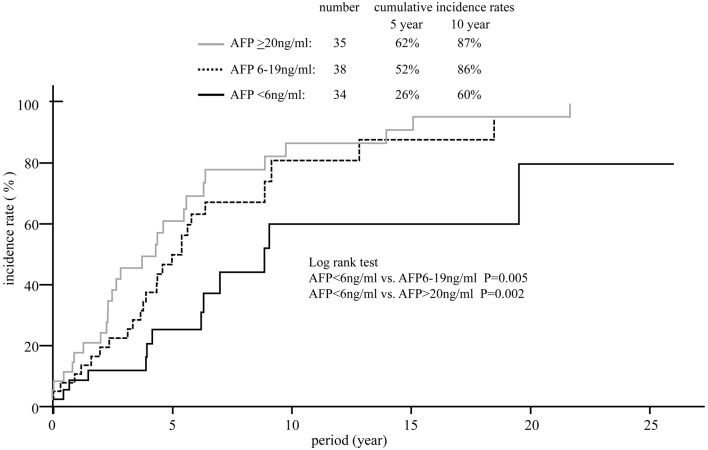
Cumulative incidence rates of hepatocellular carcinoma (HCC) according to α-fetoprotein (AFP) levels.

During the follow-up period, HCC developed in 68 (63.6%) patients. Kaplan-Meier estimates of the cumulative risk of HCC are shown in Fig. 1. The 10-year cumulative incidence rate of HCC was 80%. The cumulative incidence of HCC in patients with various AFP levels is shown in Fig. 2. The 10-year cumulative risk of HCC was 60% in the 34 patients with AFP levels <6 ng/ml at study entry, 86% in the 38 patients with AFP levels between 6 and 19 ng/ml and 87% in the 34 patients with AFP levels ≥20 ng/ml. Significant differences were observed in the HCC incidence between those with AFP level <6 ng/ml and those with an AFP level between 6 and 19 ng/ml and ≥20 ng/ml.

## Discussion

In the present study, the AFP level was identified as a risk factor for HCC in HCV patients with liver cirrhosis. Notably, patients with high (≥20 ng/ml) and elevated AFP levels (between 6 and 19 ng/ml) had an increased risk of HCC development. This deviated slightly from the serum AFP levels of healthy adults reported to range between 0.1 and 5.8 ng/ml (16). In the present study, analyses were performed by setting various AFP cut-off levels to evaluate their performance as risk factors. In HCV patients with cirrhosis, an AFP level ≥6 ng/ml was observed to be associated with the development of HCC in the multivariate analysis.

AFP is used as a serological marker of HCC and employed in combination with USG for HCC screening (17,18). Numerous studies have demonstrated an elevated AFP level to be a risk factor for the development of HCC in HCV patients (19–26). There is extensive evidence demonstrating that AFP is functionally an embryonic and fetal carrier/transport molecule for a number of of ligands, including fatty acids, bilirubin, heavy metals, steroids, retinoids, drugs, dyes and antibiotics (27). However, the biological and pathophysiological roles of the association of AFP with an increased risk of HCC development remain unclear. Tateyama *et al* reported that AFP levels were elevated in parallel with advanced fibrosis stages and correlated well with the fibrosis stage (26). Since the patients with slightly elevated AFP levels (between 6 and 19 ng/ml) had moderately advanced liver fibrosis stages, these AFP levels may indicate an elevated risk of HCC in patients with chronic HCV infection. Li *et al* identified a functional link between cytoplasmic AFP and the PTEN/AKT signalling pathway and provided further evidence for the understanding of the novel role of cytoplasmic AFP in the maintenance of tumor cell growth (28). The silencing of AFP expression by a knockdown of its gene may play a role in growth arrest and apoptosis in human HCC cells (28–31).

IFN has been used to treat patients with HCV infection. A failure to achieve an SVR with IFN-based therapies, pre-existing advanced hepatic fibrosis and/or cirrhosis are major predictors of HCC (20,32–35). Numerous Japanese cohort studies have demonstrated that IFN therapy reduces the incidence of HCC, not only in sustained virological responders but also in transient responders in whom the elimination of HCV has failed (32,36–40). In cirrhotic patients, Nishiguchi *et al* reported that the relative risk of patients receiving IFN-α treatment developing HCC was 0.067 in comparison with the control group (34). By contrast, Valla *et al* were unable to demonstrate any significant benefit for the prevention of HCC in patients with or without IFN treatment (41). Cammà *et al* suggested a slight preventive effect of IFN on HCC development in patients with HCV-related cirrhosis (42). Shiffman *et al* reported that continuous IFN therapy led to a decline in hepatic fibrosis despite the persistence of viremia (43). In addition, Nomura *et al* reported that the AFP level was significantly decreased at 3 months following the start of low-dose long-term IFN treatment (15). Murashima *et al* demonstrated that IFN therapy, but not Stronger Neo-Minophagen C (SNMC), universally reduced basic AFP levels (44). In an *in vitro* study of the effects of IFN on an HCC cell line, IFN exhibited antitumor effects (45). Taken together, these findings suggest that AFP levels may aid the prediction of the development of HCC during IFN-based treatments, including long-term low-dose IFN therapy.

In conclusion, AFP is a non-invasive predictive marker of the development of HCC in HCV patients. The present study indicates that high (≥20 ng/ml), and slightly elevated (between 6 and 19 ng/ml) AFP levels, may suggest a substantial risk of HCC development, complementing the fibrosis stage. By contrast, AFP levels <6 ng/ml indicate a low risk of HCC development.

## Figures and Tables

**Table I:** Characteristics of 107 studied hepatitis C patients with liver cirrhosis.

Characteristic	Value
Number of patients	107
Age (years), median (IQR)	62.5 (13.3)
Gender, n (%)	
Male	54 (51)
Female	53 (49)
Height (m), median (IQR)	1.58 (0.2)
Weight (kg), median (IQR)	56.4 (13.3)
BMI (kg/m^2^), median (IQR)	22.6 (4.2)
Alcohol consumption, n (%)	
Excessive	11 (10)
Not excessive	96 (90)
Diabetes mellitus, n (%)	
+	44 (41)
-	63 (59)
Diagnosis, n (%)	
Histological	80 (75)
Clinical	27 (25)
Child-Pugh grade, n (%)	
A	56 (52)
B	44 (41)
C	7 (7)
Platelet count (10^3^/μl), median (IQR)	100 (6.5)
AST (IU/l), median (IQR)	71 (64)
ALT (IU/l), median (IQR)	60 (61)
γ-GTP (IU/l), median (IQR)	45 (58)
Bilirubin (mg/dl), median (IQR)	1.0 (0.9)
Albumin (mg/dl), median (IQR)	3.8 (0.9)
TC (mg/dl), median (IQR)	152 (44)
TG (mg/dl), median (IQR)	92 (57
AFP (ng/ml), median (IQR)	11 (24)
<6, n (%)	34 (32)
6–19, n (%)	38 (35)
≥20, n (%)	35 (33)
BCAA, n (%)	
+	39 (36)
-	68 (64)
Interferon therapy, n (%)	
SVR	11 (10)
Non-SVR	32 (30)
No therapy	64 (60)

Data are median (IQR) or frequency (%). BMI, body mass index; AST, aspartate transaminase; ALT, alanine transaminase; TC, total cholesterol; TG, triglyceride; BCAA, branched-chain amino acids; AFP, α-fetoprotein; SVR, sustained virological response.

**Table II:** Factors increasing the risk of hepatocellular carcinoma (HCC) determined by univariate analysis.

Parameters	Hazard ratio	P-value
Age (years)		
>62	1.29	0.291
Gender		
Male	0.80	0.360
BMI (kg/m^2^)		
>25	0.88	0.636
Alcohol consumption		
Excessive	1.40	0.211
Diabetes mellitus (%)		
+	1.10	0.712
Child-Pugh grade		
A	1	-
B	1.20	0.474
C	0.94	0.925
Platelet (10^3^/μl)		
<100	1.07	0.788
AST (IU/l)		
≥71	1.83	0.016
ALT (IU/l)		
≥60	1.80	0.020
γ-GTP (IU/l)		
≥45	1.25	0.970
Bilirubin (mg/dl)		
≥1.0	0.72	0.189
Albumin (mg/dl)		
<3.8	0.85	0.520
TC (mg/dl)		
≥152	0.66	0.095
TG (mg/dl)		
≥92	0.76	0.269
AFP (ng/ml)		
<6	1	-
6–19	2.54	0.006
≥20	2.71	0.003
BCAA		
+	1.59	0.063
Interferon therapy (%)		
No therapy	1	-
Non-SVR	0.77	0.366
SVR	0.26	0.031

BMI, body mass index; AST, aspartate transaminase; ALT, alanine transaminase; TC, total cholesterol; TG, triglyceride; BCAA, branched-chain amino acids; AFP, α-fetoprotein; SVR, sustained virological response.

**Table III:** Factors increasing the risk for hepatocellular carcinoma (HCC), determined by multivariate analysis.

Parameters	Hazard ratio	95% CI	P-value
AST (IU/l)			
≥71	1.27	0.72–2.26	0.411
ALT (IU/l)			
≥60	1.40	0.81–2.43	0.229
AFP (ng/ml)			
<6	1	-	-
6–19	2.22	1.13–4.38	0.006
≥20	2.09	1.03–4.23	0.003
Interferon therapy (%)			
No therapy	1	-	-
Non-SVR	0.99	0.55–1.80	0.989
SVR	0.46	0.14–1.57	0.218

CI, confidence interval; AST, aspartate transaminase; ALT, alanine transaminase; AFP, α-fetoprotein; SVR, sustained virological response.

## References

[1] El-Serag HB, Mason AC (2000). Risk factors for the rising rates of primary liver cancer in the United States. Arch Intern Med.

[2] El-Serag HB (2001). Epidemiology of hepatocellular carcinoma. Clin Liver Dis.

[3] El-Serag HB, Hampel H, Yeh C, Rabeneck L (2002). Extrahepatic manifestations of hepatitis C among United States male veterans. Hepatology.

[4] El-Serag HB (2002). Hepatocellular carcinoma and hepatitis C in the United States. Hepatology.

[5] El-Serag HB (2002). Hepatocellular carcinoma: an epidemiologic view. J Clin Gastroenterol.

[6] Hassan MM, Frome A, Patt YZ, El-Serag HB (2002). Rising prevalence of hepatitis C virus infection among patients recently diagnosed with hepatocellular carcinoma in the United States. J Clin Gastroenterol.

[7] El-Serag HB, Rudolph KL (2007). Hepatocellular carcinoma: epidemiology and molecular carcinogenesis. Gastroenterology.

[8] Kiyosawa K, Tanaka E (2002). Characteristics of hepatocellular carcinoma in Japan. Oncology.

[9] McGlynn KA, Tsao L, Hsing AW, Devesa SS, Fraumeni JF (2001). International trends and patterns of primary liver cancer. Int J Cancer.

[10] Bosch FX, Ribes J, Díaz M, Cléries R (2004). Primary liver cancer: worldwide incidence and trends. Gastroenterology.

[11] Hamasaki K, Nakata K, Tsutsumi T (1993). Changes in the prevalence of hepatitis B and C infection in patients with hepatocellular carcinoma in the Nagasaki Prefecture, Japan. J Med Virol.

[12] Kato Y, Nakata K, Omagari K (1994). Risk of hepatocellular carcinoma in patients with cirrhosis in Japan. Analysis of infectious hepatitis viruses. Cancer.

[13] Shiratori Y, Shiina S, Imamura M (1995). Characteristic difference of hepatocellular carcinoma between hepatitis B- and C- viral infection in Japan. Hepatology.

[14] Shiratori Y, Shiina S, Zhang PY (1997). Does dual infection by hepatitis B and C viruses play an important role in the pathogenesis of hepatocellular carcinoma in Japan?. Cancer.

[15] Nomura H, Kashiwagi Y, Hirano R (2007). Efficacy of low dose long-term interferon monotherapy in aged patients with chronic hepatitis C genotype 1 and its relation to alpha-fetoprotein: A pilot study. Hepatol Res.

[16] Taketa K (1990). Alpha-fetoprotein: reevaluation in hepatology. Hepatology.

[17] Di Bisceglie AM (1997). Hepatitis C and hepatocellular carcinoma. Hepatology.

[18] Sherman M (2005). Hepatocellular carcinoma: epidemiology, risk factors, and screening. Semin Liver Dis.

[19] Rodríguez-Díaz JL, Rosas-Camargo V, Vega-Vega O (2007). Clinical and pathological factors associated with the development of hepatocellular carcinoma in patients with hepatitis virus-related cirrhosis: a long-term follow-up study. Clin Oncol (R Coll Radiol).

[20] Bruix J, Sherman M, Practice Guidelines Committee (2005). American Association for the Study of Liver Disease: Management of hepatocellular carcinoma. Hepatology.

[21] Colombo M, de Franchis R, Del Ninno E (1991). Hepatocellular carcinoma in Italian patients with cirrhosis. N Engl J Med.

[22] Tsukuma H, Hiyama T, Tanaka S (1993). Risk factors for hepatocellular carcinoma among patients with chronic liver disease. N Engl J Med.

[23] Oka H, Tamori A, Kuroki T, Kobayashi K, Yamamoto S (1994). Prospective study of alpha-fetoprotein in cirrhotic patients monitored for development of hepatocellular carcinoma. Hepatology.

[24] Ganne-Carrié N, Chastang C, Chapel F (1996). Predictive score for the development of hepatocellular carcinoma and additional value of liver large cell dysplasia in Western patients with cirrhosis. Hepatology.

[25] Sangiovanni A, Colombo E, Radaelli F (2001). Hepatocyte proliferation and risk of hepatocellular carcinoma in cirrhotic patients. Am J Gastroenterol.

[26] Tateyama M, Yatsuhashi H, Taura N (2011). Alpha-fetoprotein above normal levels as a risk factor for the development of hepatocellular carcinoma in patients infected with hepatitis C virus. J Gastroenterol.

[27] Mizejewski GJ, Dias JA, Hauer CR, Henrikson KP, Gierthy J (1996). Alpha-fetoprotein derived synthetic peptides: assay of an estrogen-modifying regulatory segment. Mol Cell Endocrinol.

[28] Li M, Li H, Li C (2009). Alpha fetoprotein is a novel protein-binding partner for caspase-3 and blocks the apoptotic signaling pathway in human hepatoma cells. Int J Cancer.

[29] Li M, Li H, Li C (2011). Alpha-fetoprotein: a new member of intra-cellular signal molecules in regulation of the PI3K/AKT signaling in human hepatoma cell lines. Int J Cancer.

[30] Yang X, Zhang Y, Zhang L, Zhang L, Mao J (2008). Silencing alpha-fetoprotein expression induces growth arrest and apoptosis in human hepatocellular cancer cell. Cancer Lett.

[31] Li M, Li H, Li C (2009). Cytoplasmic alpha-fetoprotein functions as a co-repressor in RA-RAR signaling to promote the growth of human hepatoma Bel 7402 cells. Cancer Lett.

[32] Yoshida H, Shiratori Y, Moriyama M (1999). Interferon therapy reduces the risk for hepatocellular carcinoma: national surveillance program of cirrhotic and noncirrhotic patients with chronic hepatitis C in Japan. IHIT Study Group Inhibition of Hepatocarcinogenesis by Interferon Therapy. Ann Intern Med.

[33] Fattovich G, Stroffolini T, Zagni I, Donato F (2004). Hepatocellular carcinoma in cirrhosis: incidence and risk factors. Gastroenterology.

[34] Nishiguchi S, Kuroki T, Nakatani S (1995). Randomised trial of effects of interferon-alpha on incidence of hepatocellular carcinoma in chronic active hepatitis C with cirrhosis. Lancet.

[35] Yu ML, Huang CF, Dai CY, Huang JF, Chuang WL (2007). Long-term effects of interferon-based therapy for chronic hepatitis C. Oncology.

[36] Imai Y, Kawata S, Tamura S (1998). Relation of interferon therapy and hepatocellular carcinoma in patients with chronic hepatitis C. Osaka Hepatocellular Carcinoma Prevention Study Group. Ann Intern Med.

[37] Kasahara A, Hayashi N, Mochizuki K (1998). Risk factors for hepatocellular carcinoma and its incidence after interferon treatment in patients with chronic hepatitis C. Osaka Liver Disease Study Group. Hepatology.

[38] Ikeda K, Saitoh S, Arase Y (1999). Effect of interferon therapy on hepatocellular carcinogenesis in patients with chronic hepatitis type C: A long-term observation study of 1,643 patients using statistical bias correction with proportional hazard analysis. Hepatology.

[39] Okanoue T, Itoh Y, Kirishima T (2002). Transient biochemical response in interferon therapy decreases the development of hepatocellular carcinoma for five years and improves the long-term survival of chronic hepatitis C patients. Hepatol Res.

[40] Hino K, Okita K (2004). Interferon therapy as chemoprevention of hepatocarcinogenesis in patients with chronic hepatitis C. J Antimicrob Chemother.

[41] Valla DC, Chevallier M, Marcellin P (1999). Treatment of hepatitis C virus-related cirrhosis: a randomized, controlled trial of interferon alpha-2b versus no treatment. Hepatology.

[42] Cammà C, Di Bona D, Craxì A (2004). The impact of antiviral treatments on the course of chronic hepatitis C: an evidence-based approach. Curr Pharm Des.

[43] Shiffman ML, Hofmann CM, Contos MJ (1999). A randomized, controlled trial of maintenance interferon therapy for patients with chronic hepatitis C virus and persistent viremia. Gastroenterology.

[44] Murashima S, Tanaka M, Haramaki M (2006). A decrease in AFP level related to administration of interferon in patients with chronic hepatitis C and a high level of AFP. Dig Dis Sci.

[45] Yano H, Iemura A, Haramaki M (1999). Interferon alpha receptor expression and growth inhibition by interferon alpha in human liver cancer cell lines. Hepatology.

